# Integrating mHealth and Systems Science: A Combination Approach to Prevent and Treat Chronic Health Conditions

**DOI:** 10.2196/mhealth.4150

**Published:** 2015-06-02

**Authors:** Nicolas Michel Oreskovic, Terry T Huang, Jon Moon

**Affiliations:** ^1^Division of General Academic PediatricsDepartment of PediatricsMassachusetts General HospitalBoston, MAUnited States; ^2^Department of PediatricsHarvard Medical SchoolBoston, MAUnited States; ^3^School of Public HealthCity University of New YorkNew York, NYUnited States; ^4^MEI Research, Ltd.Edina, MNUnited States

**Keywords:** mHealth, systems science, chronic health conditions, obesity, physical activity

## Abstract

Chronic health conditions are a growing global health concern and account for over half of all deaths worldwide. Finding ways to decrease the burden of and resources allotted to chronic health conditions is of primary importance. Recent advances in technology and insights into modeling techniques offer promising approaches, which if combined, represent a novel direction that would further advance the prevention and treatment of chronic health conditions.

## Introduction

Chronic health conditions (CHC) account for over half of all deaths worldwide. In the United States, 1 in 4 adults and 1 in 15 children suffer from two or more CHC, with 86% of all health care dollars spent on the treatment of CHC [1]. Obesity alone is a major population burden, with 2 out of 3 adults and 1 out of 3 youth overweight or obese in the United States [2]. Many CHC, such as obesity, are difficult to treat due to their complex etiologies; managing such complicated multifactorial health conditions continues to tie up valuable health care resources and accounts for substantial health care costs. Finding ways to decrease the costs and resources allotted to CHC is of primary importance. Recent advances in technology and insights into the use of systems science are promising approaches. Systems science is used to understand complex connections between structure and behavior in a system over time. Mobile health technology (mHealth) and systems science are two fields that have separately been applied to address complex problems with some success. A combined mHealth and systems science approach would represent a novel direction that would further advance the prevention and treatment of CHC.

## mHealth

mHealth is increasingly recognized as a tool to manage CHC, reduce health disparities, and address complex health problems [3-5]. In part, mHealth accomplishes this by automating processes that are currently resource-heavy. mHealth describes an array of technologies that encompass wireless sensors, software, and mobile phones worn and accessed by caregivers, patients, and individuals interested in being engaged in their own health to facilitate collecting and communicating health-related data. The nearly universal presence of mHealth technology throughout most areas of health care and ubiquity of mobile phones in the population, including underserved populations, hold great promise for increasing the integration of empirical real-time data into clinical practice and expanding the reaches of health care delivery, all to the benefit of patients’ health [3,6]. mHealth technology also offers unique advantages by being able to collect objective, patient-generated sensor data, such as activity and location, that are particularly important for CHC, given the underlying importance of health habits in most CHC [7]. Beyond using location and activity data, mHealth has the added ability to adapt to changes in a user’s location and activity, to changes in the user’s environment (eg, seasons), and to adapt to users (based on continuous feedback). As mHealth offers the power to dramatically expand access to care through objective data collection, increased efficiency, and enhanced data-driven practice, the next step is to embed systems science insights from diverse fields into mHealth technology, so that we can enhance the capability of mHealth as an effective and scalable tool for the prevention and treatment of CHC. One practical example of this combined approach is in obesity care; while an abundance of obesity- and weight management-related mHealth applications have surfaced, these applications have yet to incorporate systems science approaches.

## Systems Science

While mHealth increases efficiency through engineering solutions, systems science provides a theory-based approach to improve both efficiency and effectiveness. Systems science is a particularly useful frame to understand complex relationships that are inherent in many CHC affected by biologic, social, and environmental factors [8]. A systems-oriented approach provides several key techniques to change behavior-an important factor in the outcome of most CHC-including information feedback, ownership, collaboration, competition, accountability, and rewards. A systems approach works by helping to identify which “levers” to pull, and in which order, in a series of often nonlinear complex relationships, thus allowing complicated and dynamic relationships to be understandable, actionable, and efficient while providing the optimal impact. There is growing interest in the scientific community in applying systems techniques to understand and disentangle the complex relationships and etiologies underlying CHC, exemplified in two 2010 Institute of Medicine reports, one on obesity prevention [9] and one on improving population health [10].

## mHealth-Systems Science

Both mHealth and systems science offer to advance solutions for complex CHC by streamlining efforts and focusing on the most actionable levers. The two fields are aligned naturally and offer complementary approaches to improving the delivery of CHC treatments. While using mHealth alone can impact efficiency by automating steps and scaling up reach, an approach already being employed to treat various chronic diseases including obesity, diabetes, and tobacco addiction [11-14], it is not sufficient to marshal mHealth resources to automatize complex health problems. To truly impact the morbidity and mortality associated with complex CHC, a multi-step staged approach is required, an approach capable of identifying the steps worth automating and those which can be omitted, an approach that will increase data processing and data analysis speeds, increase efficiency, and decrease human error without impacting the desired primary outcome. The starting point in this engineering approach might be to identify all the known and anticipated steps in an intervention, including data collection, data processing, and decision points [15]. A weight analysis may follow, wherein the steps that are deemed to be most salient are prioritized for automation. The exact determination of weights may vary depending on the CHC, intended treatment, and resources available. For example, importance may be placed on cost, human labor, processing time requirements, patient preference, or anticipated prevalence of uptake. Once steps have been identified and prioritized, sequential automation can ensue until all the steps which have been deemed worth automating have been automated or until available resources (eg, time, money) have been depleted. The specific sequence and methods used for each staged approach will vary depending on the CHC and the treatment being delivered; however, the common goal should be to create an automated solution that is efficient, parsimonious (using the fewest possible resources necessary to achieve a desired outcome), and tailored.

The extent of automation may be integrated or complete. In integrated automation, the goal is to enhance and facilitate human-based care; creating a complementary approach where complex and resource-heavy programs are made more efficient through automation to facilitate CHC treatments. In complete automation, the goal of automation is to remove the health care provider from the equation altogether, such that all aspects of CHC management, including data collection, integration, and analysis with feedback loops, are managed electronically. Although both scenarios are achievable, this article focuses on integrated approaches and solutions.

We recently designed, and are pilot testing, an intervention to increase physical activity in obese adolescents with behavior change counseling guided by providing the adolescents with objective information on their location and activity [16]. The intervention was proven feasible and is effective among adolescents. The heavy manual burden and various decisions surrounding processing steps and behavioral counseling options, however, illustrate the complexity of promoting healthy behavior change and reveal the study’s limited scalability in the absence of enhanced mHealth capabilities. In this case, mHealth can be expanded to automate key processing steps (eg, combining objective location and activity data) and decision points, in addition to improving communication with participants.

The capabilities of mHealth technology can be augmented by incorporating systems insights directly within it. For example, intervening directly on information and social feedbacks is a central strategy in a systems approach. Obesity has been identified as one CHC where using systems insights to tailor feedback can be particularly impactful [17]. These feedbacks can maximize successful behavior change in several ways: (1) participant ownership in a health intervention can be increased by directly allowing users to see and use their own data. In addition, ownership can be further enhanced by allowing participants to customize their mHealth application and design alternative solutions for health behavior goals; (2) collaboration can be improved by allowing study participants to be linked to their health care provider or health coach directly via their mHealth application and select whom they wish to have involved in counseling sessions (eg, family, parents, friends, health care providers, or health coaches). In addition, the technology platform can also connect participants who have similar activity goals, thus creating emergent social networks that are goal-oriented and provide additional support to participants; (3) competition can be further leveraged by allowing individuals or groups to accumulate points over time, with top performers viewable to everyone on a common mHealth application; (4) goal-setting is often used to arrive at a personalized health behavior goal, mutually agreed upon by the participant, parent, and pediatrician. Accountability can be further enhanced using ecologic momentary assessment techniques [18], by sending reminders and connecting with participants to re-enforce commitment to the program. Further, the technology platform can also allow parents, health providers or other members of participants’ online social network to directly check in with participants when noncompliance with a goal is detected. Rewards can be built into each system lever as incentives to maximize the likelihood of changing behavior. Several of these techniques, including data organization, competition, and feedback, have previously been proposed as part of a gaming approach to obesity self-management [19]. Gaming traditionally limits its focus on the use of “controlling” devices (eg, keyboard, mouse, joystick), and involves tight but limited and inflexible loops for the user. A combined systems-science mHealth approach offers the ability to broaden the approach, by also employing “monitoring” devices (eg, sensors), to collect objective patient-generated data, and feeding this data into feedback and counseling. A combined approach has the further ability to tailor feedback, providing an optimized combination of human and machine intelligence, with content and feedback means adapted to the user.

A synergistic “mHealth-systems science” approach should be part of the next-generation public health interventions for CHC. Leveraging systems insights within mHealth technology can further optimize behavior change strategies. As technology becomes ever more affordable, this combined approach offers a promising vehicle for scaling up systems-oriented behavior change interventions to the whole population.

## Abbreviations

CHC: chronic health conditions
mHealth: mobile health technology
